# Availability of HIV prevention and treatment services for people who inject drugs: findings from 21 countries

**DOI:** 10.1186/1477-7517-10-13

**Published:** 2013-08-19

**Authors:** Zaino Petersen, Bronwyn Myers, Marie-Claire van Hout, Andreas Plüddemann, Charles Parry

**Affiliations:** 1 Medical Research Council, Alcohol and Drug Abuse Research Unit, PO Box 19070, Tygerberg 7505, Cape Town, South Africa; 2 Department of Psychiatry and Mental Health, University of Cape Town, Groote Schuur Hospital, Observatory, Cape Town, South Africa; 3 School of Health Sciences; Waterford Institute of Technology, Waterford, Ireland; 4 Department of Psychiatry, University of Stellenbosch, PO Box 19063, Tygerberg 7505, Cape Town, South Africa

**Keywords:** Injecting drug use, People who inject drugs, Harm reduction, HIV prevention

## Abstract

**Background:**

About a third of the global HIV infections outside sub-Saharan Africa are related to injecting drug use (IDU), and this accounts for a growing proportion of persons living with HIV. This paper is a response to the need to monitor the state of the HIV epidemic as it relates to IDU and the availability of HIV treatment and harm reduction services in 21 high epidemic countries.

**Methods:**

A data collection form was designed to cover questions on rates of IDU, prevalence and incidence of HIV and information on HIV treatment and harm reduction services available to people who inject drugs (PWID). National and regional data on HIV infection, IDU in the form of reports and journal articles were sought from key informants in conjunction with a systematic search of the literature.

**Results:**

Completed data collection forms were received for 11 countries. Additional country-specific information was sourced via the literature search. The overall proportion of HIV positive PWID in the selected countries ranged from 3% in Kazakhstan to 58% in Vietnam. While IDU is relatively rare in sub-Saharan Africa, it is the main driver of HIV in Mauritius and Kenya, with roughly 47% and 36% of PWID respectively being HIV positive. All countries had antiretroviral treatment (ART) available to PWID, but data on service coverage were mainly missing. By the end of 2010, uptake of needle and syringe programmes (NSP) in Bangladesh, India and Slovakia reached the internationally recommended target of 200 syringes per person, while uptake in Kazakhstan, Vietnam and Tajikistan reached between 100-200 syringes per person. The proportion of PWID receiving opioid substitution therapy (OST) ranged from 0.1% in Kazakhstan to 32.8% in Mauritius, with coverage of less than 3% for most countries.

**Conclusions:**

In order to be able to monitor the impact of HIV treatment and harm reduction services for PWID on the epidemic, epidemiological data on IDU and harm reduction service provision to PWID needs to be regularly collected using standardised indicators.

## Background

Globally there are approximately 15.9 million people who inject drugs (PWID), with around 80% of PWID living in low and middle income countries (LMIC) [1]. The continued high prevalence of injection drug use (IDU) is cause for concern because of the strong association between IDU and risk for Human Immunodeficiency Virus (HIV) transmission. This association is largely due to the sharing of drug paraphernalia with other PWID, with needle sharing accounting for about one tenth of new HIV infections globally and almost a third of all new HIV infections outside sub-Saharan Africa [2]. Regions with particularly high rates of new HIV infections among PWID include Eastern Europe and Central Asia as well as East and South-East Asia [3].

In contrast, there has been a decline in HIV incidence in the USA and Western Europe [4]. This decline has been spurred on by behaviour changes among at-risk populations (including PWID) as well as improved access to HIV treatment and harm reduction services for these populations [5-7]. However, access to adequate HIV treatment and harm reduction services for PWID varies from country to country. Earlier work has shown that many of the countries with high rates of new HIV infections among PWID fail to provide PWID with access to the recommended comprehensive package of evidence-based HIV treatment and harm reduction services [8,9]. In particular, limited availability of antiretroviral therapy (ART) and harm reduction services (such as needle and syringe programmes (NSP) and opioid substitution therapy (OST)) is a major barrier to efforts to limit HIV transmission amongst PWID and their injecting and sexual partners [10-12].

Even in countries that report the availability of core HIV treatment and harm reduction services for PWID, earlier work has highlighted that ART, NSP and OST service coverage is generally below the minimum threshold needed to adequately prevent new HIV infections among PWID [12-14]. Concerns about the poor availability and limited coverage of these services have led to continued calls for the introduction of and, where present, scaling up of the provision of these services. Although these services are especially important for countries where the HIV epidemic is concentrated amongst PWID, they are also vital for countries where IDU is an emerging public health concern [11-13]. In order to guide effective country-level responses to these issues, updated information is needed on IDU rates, prevalence of HIV among PWID, the types of treatment and harm reduction services provided to PWID living with HIV and the degree of service coverage. Although global reviews of the epidemiology of HIV among PWID [2] and HIV service coverage [12] have been conducted in the past five years, several LMICs with emerging HIV epidemics among PWID have not had data available to contribute to these reviews. For other high epidemic countries, it is unclear whether the prevalence of HIV and the extent to which HIV treatment and harm reduction services are available for PWID have changed since the time of the last review [2].

This paper is in response to this need for updated data on HIV prevalence and harm reduction service provision among PWID for 21 LMICs with either high or emerging HIV epidemics among PWID. More specifically, this paper describes the (i) prevalence of IDU; (ii) the prevalence of HIV among PWID; (iii) the provision of NSP, OST and ART to PWID; and (iv) the extent to which NSP, OST and ART are provided relative to the size of the IDU population in the 21 selected countries.

## Methods

### Sample description

Twenty-one countries were chosen by the Steering Committee of the International Reference Group to the United Nations on IDU and HIV to participate in this study. These countries were selected based on the state of the HIV epidemic in these countries, resource needs and availability of partner organizations to support efforts to introduce and/or scale up HIV treatment and harm reduction services. Selected countries included: Afghanistan, Bangladesh, Belarus, China, India, Indonesia, Kenya, Kazakhstan, Kyrgyzstan, Lithuania, Mauritius, Moldova, Myanmar, Nepal, Nigeria, Pakistan, Russia, South Africa, Tanzania, Ukraine and Vietnam.

### Data collection

A data collection form was used to collect national and regional estimates of the population prevalence of IDU, prevalence of HIV, availability and coverage of HIV treatment (the provision of ART) and harm reduction services for PWID (the provision of NSP/OST in the 21 countries between June 2010 and June 2011). These forms were completed by key contacts who were experts in IDU and HIV in the selected countries. The forms were also used by the research team to guide data extraction from country reports and relevant publications. The content of this data collection form was informed by the Steering Committee of the Reference Group, the Secretariat to the UN Reference Group and prior data collection exercises conducted by the Reference Group [2,11]. In addition, key contacts were asked to provide supporting evidence in the form of reports, publications and presentations where these were available. After only receiving completed data collection forms from 11 of the 21 countries and realizing that recent 2010-2011 information on these key indicators was not yet available for many of the selected countries, we undertook a literature search using Sciencedirect, PubMed, Google, and Google scholar and Ebscohost. The websites of international agencies with a presence in these countries (e.g. UNODC, UNAIDS and WHO), and the conference proceedings of recent international meetings on HIV and drug use to identify recent reports and publications on the epidemiology of IDU and HIV in the selected 21 countries. This information was searched for between January 2011 and January 2012, and only the most updated information (from 2010 and later) was included. Literature was further sifted according to whether it contained information on the 21 selected countries and whether it described the main prompts on the data collection form (IDU and HIV prevalence, as well as OST, NSP and ART availability). Data were extracted from these reports, presentations and publications using the aforementioned data collection form. Information from the literature was checked by three staff members, with one member doing the search, and two others cross checking and updating information where necessary. Reference group members in the diferent countries were alo given the opportunity to update findings.

## Results

### Prevalence of IDU and HIV among PWID

Of the 21 participating countries, the Russian Federation and the Ukraine were countries identified with the highest rates of reported IDU, with roughly 2 million (2% of adult the population) and 425000 (1.3% of the adult population) injecting drugs respectively (Table 1). In the selected countries, the overall proportion of PWID living with HIV ranged from 3% in Kazakhstan to 58% in Vietnam (Table 1). Taking the midpoint in cases where a range was given, the average proportion of PWID living with HIV across the 19 countries where data were available was almost 22%. However country-level estimates sometimes mask regional concentrations of HIV among PWID. For example, a recent UNAIDS publication reported HIV prevalence in St Petersburg, Russia, to have doubled in the past five years, with approximately 60% of PWID in this city living with HIV [6].

**Table 1:** Estimated population size, proportion of population that inject drug and the number of HIV-positive drug users*

**Country**	**Population (all ages)**	**Population (15-64 years)**	**No of PWID**^**1 **^**(range)**	No. of PWID that are HIV+* (range)	% of HIV+ PWID**
Afghanistan	29,835,392	16,492,454	19,000-25,000	1,330-1,750	7
Bangladesh	158,570,535	96.847,950	20,000-40,000	1,600-3,200	8
Belarus	9,557,552	6,870,907	69,200-83,400	9,480-11,425	13.7
China	1,336,718,015	983,280,460	642,000	44,940-83,460	7-13
India	1,210,193,422	771,476,660	177,000-180,000	16,284-16,560	9.2
Indonesia	237.6 million	163,367,691	222,500	60,075	27
Kenya	41,070,934	22,634,399	30,000	10,800-12,900	36-43
Kazakhstan	16,455,000	11,028,216	186,000	5,580	3
Kyrgyzstan	5,587,443	3,656,055	_______	_______	_______
Lithuania	3.2 million	2,475,902	5,458	1,250	22.9
Mauritius	1,286,340	921,618	10,000	4,740	47.4
Moldova	4,314,377	3,193,494	25,000	4,450	17.8
Myanmar	53,999,804	36,442,403	75,000	27,750-28,500	37-38
Nepal	29,391,883	17,951,875	28,500	5,415	19
Nigeria	155,215,573	86,830,770	________	_______	8.9^18^
Pakistan	187,342,721	113,072,889	125,000	26,250	21
Russia	142,905,208	99,594,130	2 million	_______	_______
South Africa	49,054,031	32,259,360	67,000^1^	12,998	19.4
Tanzania	42,746,620	23,547,672	25,000-50,000	10,500-21,000	42
Ukraine	45,134,707	31,956,390	325000-425000	74,425-97,325	22.9
Vietnam	90,549,390	62,721,185	142,000	45,440-82,360	32-58

^1^ calculated from a national 2008 Survey.

*An estimation of the number of PWID that were HIV positive by 2010. Where updated data was not available the latest reported prevalence was used, including periods 2006-2010. **Calculated at the midpoint of the range where applicable. ______ Data is not available.

HIV prevalence among PWID in one region of Afghanistan was found to have increased from 3% in 2006 to 7% by 2011 (Table 1). While pilot harm reduction programmes are available (NSP’s and drug rehabilitation centres), a scale-up of these interventions is urgently required for IDU’s in order to prevent a future HIV epidemic [15,16]. Whilst Bangladesh experienced an increase in the rates of HIV between 2001 and 2009, one area (Dhaka) where harm reduction programmes are available experienced a decrease in the rates of new HIV infections. Here the availability of harm reduction programmes for PWID resulted in a reduction in HIV prevalence from 7% in 2007 to 5.3% in 2011 [6]; (Table 1).

While IDU is relatively rare in sub-Saharan Africa, IDU appears to act as the main driver of HIV in Mauritius, with roughly 47% of PWID being HIV positive. Other available research in sub-Saharan Africa shows a high HIV prevalence among PWID in Kenya (36%), Zanzibar in Tanzania (26%), and the Kano region in Nigeria (10%) [5]; (Table 1).

### Availability of core HIV treatment (ART) and harm reduction (NSP/OST) services for PWID

All 21 countries reported that ART was available to PWID living with HIV (Table 2). However, most countries collected data on the number of services providing ART to the general public rather than the number of services providing ART to PWID specifically. Of concern is the large number of countries with high prevalence rates of HIV among PWID that do not know the proportion of HIV-positive PWID receiving ART. Even where data were available, the estimated proportion of PWID living with HIV and using ART was low, ranging from 0.6% in Afghanistan to 22% in Bangladesh.

**Table 2:** Availability and coverage of ART among PWID in 2010-

**Country**	**ART availability 2011**	**Est. no. of HIV positive pop receiving ART**^**3**^	**No. of HIV+ PWID**	**Est. no. of HIV+ PWID receiving ART**
Afghanistan	yes	---------	1330-1750	12
Bangladesh	yes	1725	1600-3200^5^	353 (Dec 09)
Belarus	yes	2,614	_______	_______
China	yes	86,122	169120-314080	12 762
India	yes	424,802	16284-16560	1900
Indonesia	yes	19,572	_______	_______
Kenya	yes	432,621*	10800-12900	_______
Kazakhstan	yes	1,336	5208	182
Kyrgyzstan	yes	548	_______	115
Lithuania	yes	_______	1250	62
Mauritius	yes	646	4740	530
Moldova	yes	1,237	4450	446
Myanmar	yes	29,825	27750-28500	_______
Nepal	yes	4,867	8%	_______
Nigeria	yes	359,181P	8.9%^18^	_______
Pakistan	yes	1,892	26,250	_______
Russia	yes	79,430	________	_______
South Africa	yes	1,389,865*	1920	_______
Tanzania	yes	258,069	10500-21000	Unknown
Ukraine	yes	22,697	74425-97325	1732 (Jan 2011
Vietnam	yes	49,492	45440-82360	_______

* Estimated no of “general population” receiving ART.

______ Data are not available.

In terms of the availability of HIV prevention services for PWID, in 2011 NSPs were available in 20 of the 21 countries examined, but the number of NSP sites varied per country (Table 3). In countries where rates of IDU were low (such as South Africa) introducing NSPs is often not publicly accepted. For example one NSP site has been established in South Africa, however this service is affiliated to an organization providing services to men who have sex with men (MSM), which indicates limited reach to PWID who are not MSM. While Mauritius has some NSPs and Tanzania has pilot programmes, these services are unavailable in Kenya and Nigeria (Table 3).

**Table 3:** Availability and coverage of needle and syringe programs (NSP) in 2010-11

**Country**	**NSP availability**	**No. of NSP sites nationally**	**No. of PWID served in last 12 months**	**No. of syringes per PWID distributed in 1 year (Jan-Dec 2010)**
Afghanistan	yes	9	_______	34.7
Bangladesh	yes	120	26000	214.4
Belarus	yes	_______	_______	46.5
China	yes	1052	39,500	18.9
India	yes	261	135000	228.2
Indonesia	yes	_______	_______	10.2
Kenya	no	_______	_______	_______
Kazakhstan	yes	168	73,252	176.4
Kyrgyzstan	yes	_______	_______	_______
Lithuania	yes	12	429	_______
Mauritius	yes	52	6000 p/y	51.9
Moldova	yes	29	_______	65.8
Myanmar	Yes	40 (2008)	13368	91.7
Nepal	Yes	9	_______	56.5
Nigeria	No	_______	_______	_______
Pakistan	Yes	15	1500	_______
Russia	Yes	69	_______	_______
South Africa	Yes	1	_______	_______
Tanzania	Yes	1 (new , Jul2011)	1048	_______
Ukraine	Yes	1633	174796	62.4
Vietnam	Yes	_______	_______	140.6

______ Data are not available.

In addition, information on the degree of service coverage provided by available NSPs (in terms of the total number of needles distributed per annum and the number of needles provided to each PWID) was unavailable for 9 of the 21 countries (Table 3). For the 13 countries with available data on the number of needles distributed per year, most countries (10) reported low levels of NSP coverage (Table 3). In other words, they provide fewer than 100 needles and syringes per PWID per year [17]. However by the end of 2010, NSP coverage in two countries (Bangladesh and India) had reached the internationally recommended target of 200 syringes per PWID with coverage in Kazakhstan and Vietnam having reached medium levels; that is between 100-200 syringes per PWID per year [18].

OST as treatment for opioid dependence and as a means of reducing risky injection practices was available in 17 of the 21 selected countries (Table 4). Of the countries that provided OST, methadone maintenance therapy (MMT) was recorded as the most widely used treatment modality.

**Table 4:** Availability and coverage of opioid substitution therapy (OST) 2010-11

**Country**	**OST availability**	**No. of OST sites nationally**	**MMT/BMT**	**% of PWID receiving OST in last 12 months, Dec 2010**
Afghanistan	Yes	_______	_______	0.4
Bangladesh	Yes	1	MMT	0.3
Belarus	Yes	_______	_______	0.9
China	Yes	928	MMT	28.4
India	Yes	65	BMT	3
Indonesia	Yes	9	_______	2.4
Kenya	_______	_______	_______	_______
Kazakhstan	Yes	10	MMT	0.1
Kyrgyzstan	_______	_______	_______	_______
Lithuania	Yes	21	MMT&BMT	_______
Mauritius	Yes	16	MMT	32.8
Moldova	Yes	8	MMT	1.4
Myanmar	Yes	10	MMT	1.5
Nepal	yes	8	_______	1.2
Nigeria	yes	_______	_______	_______
Pakistan	No	_______	_______	_______
Russia	No	_______	_______	_______
South Africa	Yes	_______	BMT&MMT	_______
Tanzania	Yes	1 new Feb 2011	MMT	0.7
Ukraine	Yes	125	MMT and BMT	2.1
Vietnam	Yes	8	_______	1.3

_______Data are not available.

Information on OST provision was not available for six of the 21 countries (Afghanistan, Belarus, Indonesia, Kenya, Kyrgyzstan, and Vietnam). Two countries (Russia and Pakistan) reporting no OST service provision, and with either MMT or buprenorphine maintenance therapy (BMT) available in 13 countries (Table 4). Furthermore, OST service coverage is generally poor in most of the countries reporting OST sites, with less than 3% of PWID in 11 of the 21 countries with available data. For example, South Africa has had BMT available for a few years in the private sector and recently MMT has also become available in this sector, but currently there are no public OST sites available. More specifically, the proportion of PWID receiving OST ranges from 0.1% in Kazakhstan to 32.8% in Mauritius. In addition, two countries (Bangladesh and Tanzania) have only recently started providing OST, with one pilot site available in each country.

## Discussion

This paper details the prevalence of IDU and HIV, the availability of HIV treatment (ART) and harm reduction (NSP/OST) services across 21 countries with either high or emerging HIV epidemics among PWID, and efforts to scale up service coverage to PWID. The increasing rates of IDU and HIV among IDU remain concerning. Varying rates of HIV prevalence among PWID and varying levels of HIV treatment and harm reduction service coverage have been noted.

First, this study found that countries surveyed vary with regards to IDU trends, with some having a very high prevalence of IDU, while it is an emerging trend in others. Specifically, across the 21 countries surveyed, the Russian Federation and the Ukraine are the countries with the highest prevalence of IDU, while most African countries have comparatively lower proportions of PWID. Despite this, IDU is a cause for concern in African countries, which already have generalized HIV epidemics. Failure to address IDU and HIV risk among PWID in this region will impact negatively on efforts to prevent new HIV infections and curtail the epidemic. Between one fifth and a quarter of PWID across the selected countries are living with HIV. The average proportion of PWID who are HIV positive is just under 22% across the 19 countries where data were available.

Second, the paper also highlights the poor coverage of ART services for PWID, across the 21 countries. The proportion of PWID who are HIV positive and who receive ART is very low (less than 12% with the exception of Bangladesh). This could be because they are still healthy (CD4 counts that are still high), however, this information is lacking and in light of recent reports [19], it is more likely that it is because of barriers to accessing ART for PWID. The lack of updated data with regards to IDU and HIV services to PWID is a matter of concern for many of the 21 countries. A large number of countries with high prevalence rates of HIV among PWID are not aware of the proportion of HIV positive PWID who are receiving ART. This is a major concern as ART use can help decrease risk of transmission among PWID and also their sexual partners. Data on ART service coverage disaggregated by IDU is increasingly necessary, especially in countries with a high prevalence of HIV among IDU, but also for countries where IDU is an emerging problem. This data could serve as a baseline for informing the development, implementation and scaling up of HIV treatment services for PWID.

Third, we noted that these 21 countries also vary in the extent to which they provide harm reduction services to PWID, with some countries having established harm reduction services for PWID, and others only beginning to pilot harm reduction programs, due to the rising prevalence of IDU. NSP, in particular, is a fairly new phenomenon in many of the selected countries, with some countries only having one or two pilot NSP sites to date. While most of the selected countries now have NSP available, the extent to which PWID have access to such programmes is questionable. In countries where NSPs are available, only two out of the 21 countries (Kazakhstan and Vietnam) provided medium coverage (between 100-200 syringes per IDU per year), and only two countries provided high coverage (Bangladesh and India). These findings suggest that much more needs to be done to bring these NSPs to scale and help prevent new HIV infections. Barriers to the provision of clean syringes to PWID and uptake of services also need to be addressed in order to improve harm reduction practices.

While many of the selected countries had OST available to PWID, the number of sites per country was low with very few PWID having access to such services. While not all PWID (particularly those injecting amphetamine type stimulants (ATS) require OST, OST coverage still appears to be low (less than 3%) although some exceptions were noted, namely China and Mauritius. A recent report also noted the emergence of amphetamine type stimulants (ATS) in many countries, and the need for harm reduction services for people who inject ATS [20]. This report highlights the lack of information on ATS, as most of the countries do not routinely differentiate between amphetamine and opioid injection. This lack of knowledge affects service planning, as countries have no baseline information that can be used to assess the effectiveness of efforts to scale up services.

The overriding limitation of these data is that data across countries are not collected in a uniform way, with updated information available for some countries only. Eleven of the 21 countries provided data by completing the data collection form, but even for these countries there were many gaps in terms of programmatic evidence, simply because such data are not readily available. And, while the latest progress report on the Global HIV/AIDS response indicates a dramatic improvement in evidence-based HIV prevention, even in this report the ‘evidence’ for many countries is either lacking or fragmented. This data collecting exercise whilst highlighting the lack of specific HIV-related harm reduction services for PWID in some countries, offers a benchmark for improving service coverage in response to emerging injecting trends and rates of HIV.

## Conclusion

Despite some limitations, this paper points to the need for countries, through UNODC offices or other agencies such European Monitoring Centre for Drugs and Drug Addiction (EMCDDA), to routinely collect timely data on IDU and non-IDU trends and related HIV rates using a standard data collection form and indicators, with similar time-frames for all countries. PWID remain a high risk cohort for new HIV transmissions. Standardised and routine data collection on IDU and HIV would allow policy makers, researchers and programme planners to monitor the impact of scaling up HIV treatment (ART) and harm reduction (NSP/OST) services for PWID. It remains evident that such services should be widely available to PWID.

## Abbreviations

ART: Antiretroviral Therapy; ATS: Amphetamine Type Stimulants; HIV: Human Immunodeficiency Virus; IDU: Injection Drug Use; MSM: Men who have sex with men; NSP: Needle and Syringe Programmes; OST: Opioid Substitution Therapy; PWID: People Who Inject drugs.

## Competing interests

The authors declare that they have no competing interests.

## Authors’ contribution

ZP, BM and AP conducted the research, with expert advice from CP. MvH conducted a literature search and was also involved in updating information while the paper was drafted. The forms were designed by the Secretariat to the United Nations on HIV and injection drug use (ZP, BM, CP, and AP) and the reference group. ZP wrote the first draft of the paper, with BM, MvH, AP and CP providing revisions. All authors approved the final manuscript.
